# Simultaneous ipsilateral hip and knee dislocation: Management and outcome – A case report

**DOI:** 10.1016/j.tcr.2024.101079

**Published:** 2024-07-15

**Authors:** Christian G. Falgons, Stephen J. Warner

**Affiliations:** Investigation performed at McGovern Medical School at UTHealth Houston, United States of America

## Abstract

**Case:**

This clinical case report presents a 40-year-old male who sustained an ipsilateral hip and knee dislocation with ipsilateral femoral head fracture and incomplete femoral neck fracture following a motorcycle collision.

**Conclusion:**

This report describes acute and later definitive orthopedic care and management, with focus on urgent interventions and timing of immediate treatments. Given the presented patient's favorable clinical outcomes, return to baseline activities, and absence of significant sequelae following injury, the considerations from the acute management and surgical planning of this patient's injuries can be used as a reference for treating the rare injury of ipsilateral knee and hip dislocations.

## Introduction

Isolated hip and knee dislocations are severe orthopedic injuries that, when considered separately are not uncommon. However, the simultaneous occurrence of these injuries is an exceedingly rare scenario, presenting an intricate challenge for proper management. The established treatment approach for each of these injuries is markedly influenced when they occur concurrently, necessitating prompt and well-coordinated management to achieve optimal outcomes. An extensive literature review revealed only 15 described cases of this injury [[1], [2], [3], [4], [5], [6], [7], [8], [9], [10], [11], [12], [13], [14], [15]]. Outcomes of this injury range from a relative return to baseline activities to amputation of the lower extremity. We present a complex case of an ipsilateral hip and knee dislocation, focusing on the initial management, surgical planning and rationales, and rehabilitation progress along with the patient's functional outcomes.

## Statement of informed consent

The authors certify that they have obtained appropriate patient consent, indicating that data concerning his case would be submitted for publication.

## Case report

A 40-year-old male presented to a level-one trauma center after being involved in a high-speed motorcycle collision. He sustained a right knee dislocation and ipsilateral posterior hip dislocation with right femoral head fracture and incomplete right femoral neck fracture. Other orthopedic injuries included a left medial malleolus fracture, left clavicle fracture, and a cervical spine transverse process fracture. He also sustained bilateral rib fractures, hyoid bone and thyroid cartilage fractures, and right pneumothorax.

The patient had no pertinent medical comorbidities; only a previous left knee anterior cruciate ligament (ACL) reconstruction following a sports-related injury.

Upon arrival, the patient complained of right knee and hip pain and chest pain. Physical exam revealed external rotation of the right lower extremity, tenderness to palpation of the right hip and knee, soft and compressible compartments, and a normal neurovascular exam. Due to the likelihood of intrabdominal and intrathoracic injury seen with this mechanism of injury, the patient underwent whole body computed tomography (CT) with angiography.

**Fig. 1:**
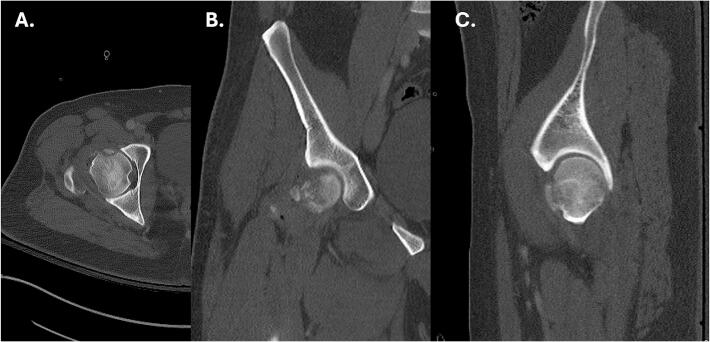
CT of the right hip demonstrating axial (A), coronal (B), and sagittal (C) views revealing a right femoral head fracture.

**Fig. 2:**
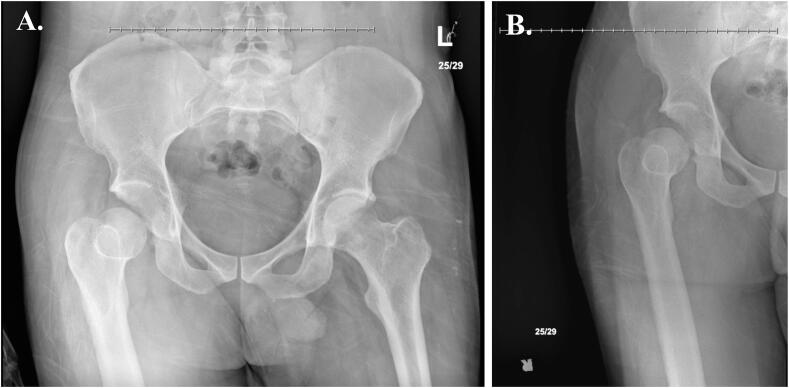
AP radiographic views of the (A) bilateral and (B) right hip demonstrating right hip posterior dislocation at initial presentation.

**Fig. 3:**
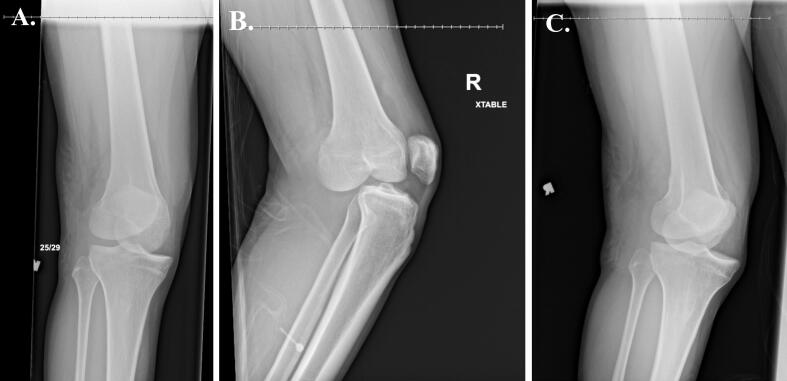
Radiographs of the right knee demonstrating (A) AP, (B) lateral, and (C) oblique views of the right knee dislocation at initial presentation demonstrating an anterior dislocation.

Radiographs and CT of the right hip revealed posterior dislocation, an acute minimally displaced Pipkin Type III fracture of the anterior right femoral head, and fracture fragments within the hip joint [16] (Fig. 1, Fig. 2). Radiographs of the right knee demonstrated an anterolateral rotational dislocation as described by the Kennedy classification [17] (Fig. 3).

Following completion of all imaging and lab tests, the patient was seen by trauma surgery for his intrathoracic injuries, stabilized by neurosurgery in a cervical collar, and placed on continuous respiratory monitoring by otolaryngology. During these evaluations, patient pain was monitored using the defense and veterans pain rating scale intermittently and well controlled with 4 mg of intravenous morphine and 100 micrograms of intravenous fentanyl.

On the same evening of arrival, four hours after the initial presentation and following medical clearance from the consulting physicians, the patient underwent urgent reductions in the emergency department with procedural anesthesia, using 70 mg of ketamine and 20 mg of propofol. The right knee was first reduced using gentle axial traction. After adequate reduction of the right knee, a 2 mm traction pin was placed in the distal femur. The patient's right hip was then reduced with traction, hip flexion, and internal rotation. Fifteen pounds of traction were applied to the distal femur. Interval reduction was confirmed by radiographs, and the right lower extremity remained neurovascularly intact, as confirmed by exam and ankle-brachial index.

**Fig. 4:**
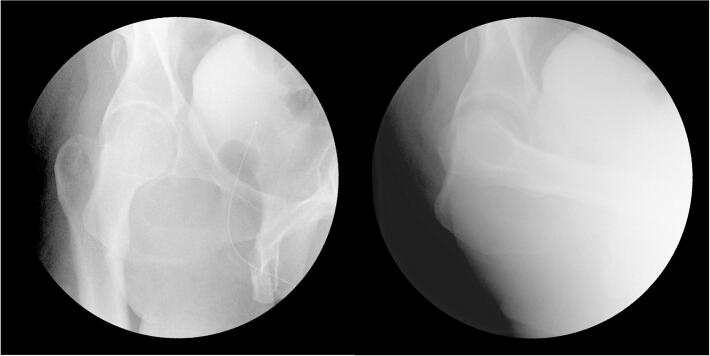
Intraoperative fluoroscopic radiographs taken during the EUA of the right hip demonstrating instability of the right hip on the obturator oblique view with the hip in approximately 60 degrees of flexion.

**Fig. 5:**
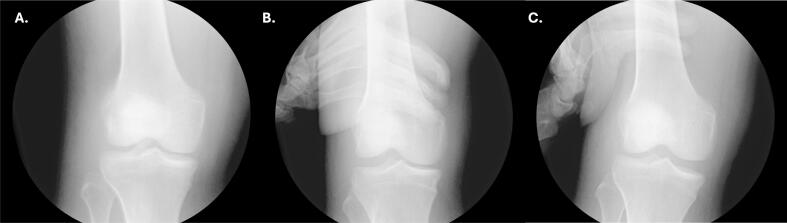
AP radiographs of the right knee during EUA without applied stress (A), applied varus stress (B), and applied valgus stress (C).

**Fig. 6:**
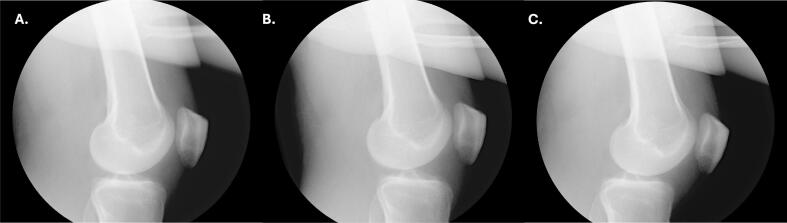
Lateral radiographs of the right knee during EUA without applied stress (A), with anteroposterior stress applied demonstrating anterior translation of the tibia (B), and with anteroposterior stress applied demonstrating posterior translation of the tibia (C). views of the right knee at the final follow-up visit, ten months after the injury.

The following morning the patient was taken directly to the operating room (OR) from the emergency department for operative fixation of his left ankle. In the OR, to obtain a more reliable exam and assess the need for further imaging or surgical intervention, he simultaneously underwent an exam under anesthesia (EUA) of the right hip and knee. Posterior instability of the right hip was noted on the obturator oblique view when it was placed in about 60 degrees of flexion (Fig. 4). The knee was found to be unstable with anterior and posterior stress on anteroposterior and lateral views (Fig. 5, Fig. 6).

The results of the EUA with the associated femoral head fracture indicated the patient for operative management of the right hip and potential immediate surgical treatment of the right knee. Given the patient's other concomitant traumatic injuries and to avoid prolonged operative time, the decision was made to proceed with operative fixation of the left ankle and delay surgical management of the right lower extremity until further imaging was obtained to establish further the need for potential surgery of the right knee and assess the degree of injury for fixation of the right hip. Femoral traction was removed from the right lower extremity and provisionally placed in a knee immobilizer as a concentric reduction of the knee could be maintained. The patient was admitted to the surgical intermediate care unit (SIMU).

**Fig. 7:**
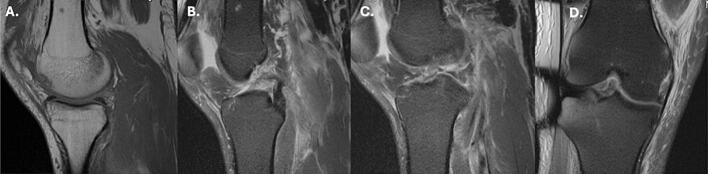
MRI of the right knee demonstrating T1 weighted sagittal view of an osteochondral fracture along the anterior portion of the medial condyle (A), T2 weighted fat-suppressed sagittal views of the complete avulsion of the PCL from its femoral attachment (B) and complete disruption of the ACL (C), and T2 weighted fat-suppressed coronal view of the complete ACL tear (D).

Magnetic Resonance Imaging (MRI) of the right knee revealed a type V knee dislocation according to the Schenck Classification [18]. Injuries included lateral patella subluxation with lateral facet cartilage delamination, lateral and medial meniscal tears, full-thickness proximal ACL tear, avulsion of the posterior cruciate ligament (PCL) from the femoral attachment, posterior joint capsule injury, posterior oblique ligament and medial retinaculum injuries, and a subchondral trabecular fracture of the anterior weightbearing aspect of the medial femoral condyle (Fig. 7). The lateral and medial collateral ligaments remained intact.

**Fig. 8:**
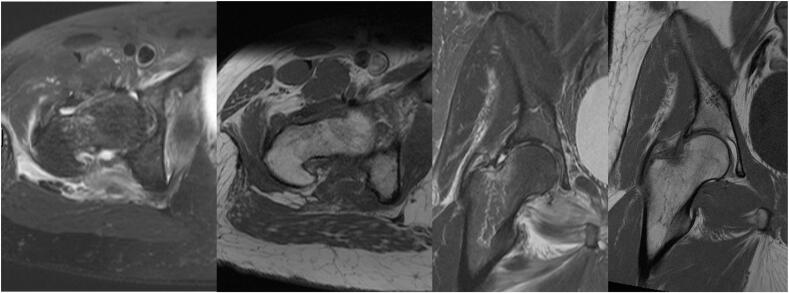
MRI of the right hip demonstrating T1 weighted (A and C) and T2 weighted (B and D) axial and coronal views, revealing an incomplete femoral neck fracture with an associated high attenuated signal representing acute inflammation.

MRI of the right hip revealed a femoral head fracture involving a 2.0 × 2.5 cm area of the anterior femoral head, impaction along the superolateral right femoral neck, and a Type I Garden Classification incomplete right femoral neck fracture [19] (Fig. 8).

Two days after admission, the patient returned to the OR for surgical management of the right hip. The right femoral neck was addressed first with three 7.0 mm cannulated screws through a lateral incision over the proximal femur. The femoral head and hip joint were then addressed using a Smith-Petersen approach, given the anterior nature of the femoral head fracture. Tenotomy of the rectus femoris and subsequent capsulotomy revealed a 3 cm area of chondral impaction on the superolateral femoral head. The hip joint was distracted, and two intra-articular fragments of less than 1 cm in size not amenable to fixation were removed. Without surgical dislocation of the hip, the femoral head fracture was reduced and stabilized with three 2.0 mm cortical screws in a countersunk fashion.

Four days after admission, the patient underwent ORIF of the left clavicle for increased stability of the upper extremities to assist in lower extremity transfers. On the tenth day of admission, after a satisfactory evaluation by a speech-language pathologist for his neck injuries, he was discharged in a hinged knee brace at 20–30 degrees of flexion with touchdown weightbearing (TDWB) status and posterior hip precautions to an inpatient rehabilitation facility for a continued comprehensive physical therapy program focused on achieving independence with functional transfers and neuromuscular reeducation. The patient was prescribed 50 mg of indomethacin taken twice a day for six weeks for heterotopic ossification prophylaxis of the right hip. The patient had an uneventful postoperative recovery and was advanced to partial progressive weightbearing on the right lower extremity at his six-week postoperative visit.

The right knee remained in a locked knee brace to protect the patient's knee when mobilizing, and full ROM of the right knee was achieved. Three months after the initial injury, he underwent open treatment of right knee osteochondral fracture of the distal femur medial femoral condyle, ACL reconstruction with quadriceps tendon autograft, and arthroscopic medial and lateral meniscus repairs.

**Fig. 9:**
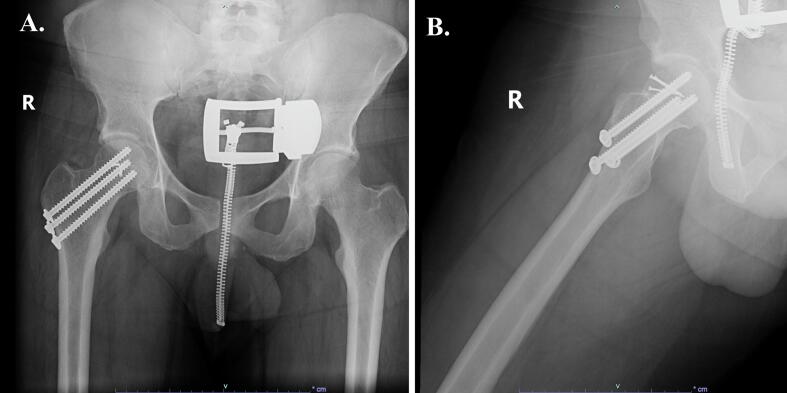
Radiographs of the right hip demonstrating (A) AP and (B) lateral views of the right hip at the final follow-up visit, ten months after the injury.

**Fig. 10:**
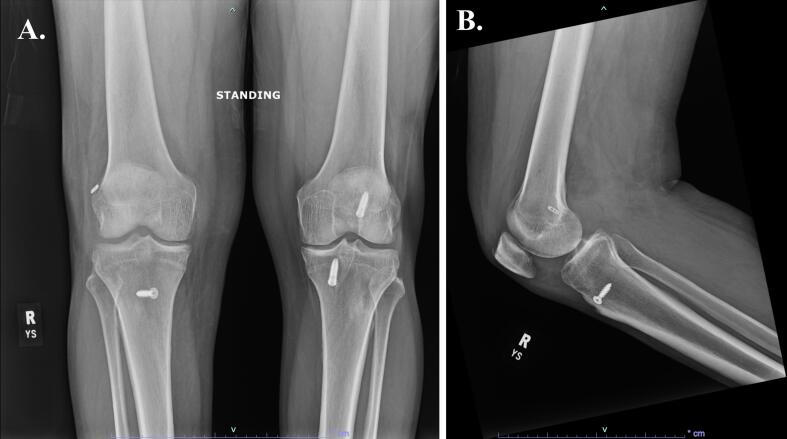
Radiographs of the right knee demonstrating (A) bilateral standing AP and (B) lateral.

Eight months after his injury, the patient had no symptoms of pain or instability in the right hip or knee on physical exam. He ambulated independently without an assistive device. He had full range of motion at the right hip and knee, had progressed to single right leg press of 160 pounds, and improved his lower extremity functional status (LEFS) score by 9 points indicating improved tolerance for his activities of daily living. Last seen at 13 months of follow-up, the patient has returned to his functional baseline and is not limited in his daily activities. Radiographs of the right hip and knee demonstrate stable alignment with a concentric reduction and no signs of posttraumatic arthritis or aseptic necrosis (Fig. 9, Fig. 10).

## Discussion

The simultaneous occurrence of an ipsilateral hip and knee dislocation is an exceedingly rare event. To the best of our knowledge, this is the sixteenth reported case in the literature. The scarcity of such cases has resulted in a lack of clear guidance for effective management strategies. Our presentation of a successfully treated case with favorable clinical outcomes aims to contribute to a more standardized approach to managing the timing and order of interventions for this rare injury.

Among the sixteen cases reviewed, thirteen describe posterior hip dislocations, and only two instances contain anterior knee dislocations, including our case. The most common pattern involved posterior hip dislocation with a posterior wall acetabular fracture (6 cases), and posterior knee dislocation. Our case was unique because it was the only case involving a posterior hip dislocation with an associated anterior knee dislocation. Most of these injuries were attributed to high-velocity motor vehicle collisions (14 cases).

An exceptional aspect of our case, never previously described, was the involvement of the femoral neck and femoral head at the hip. Isolated involvement of the femoral head was noted in two prior cases, while isolated femoral neck involvement was observed in one prior case. Due to the high energy mechanisms of these injuries, we recommend a rapid sequence MRI to be obtained of the femoral neck which has been shown to be effective in finding incomplete femoral neck fractures in other high energy injuries [20].

The most common yet most critical early complication involved vascular compromise of the popliteal artery in four patients, three resulting in popliteal artery injuries. While one patient with popliteal artery injury underwent successful arterial repair, two others required amputation. The incidence of vascular injury requiring intervention in knee dislocations is reported to be about 5.63 % [21]. The observed 18.8 % incidence of vascular injury in this small cohort with ipsilateral knee and hip dislocations is likely attributable to the forcefulness of the injuries necessary to cause this specific constellation of injuries. Additionally, common peroneal nerve palsies were observed in four patients, but notably, all resolved without intervention.

Various techniques have been employed to reduce the hip in cases of ipsilateral hip and knee dislocation. Urgent reduction of the hip upon the patient's arrival should be attempted to reduce time the hip has been dislocated. Minimal maneuvers should be performed to reduce the hip, applying axial traction to the distal femur, while simultaneously stabilizing the knee to prevent added neurovascular or ligamentous injury.

In the cases reviewed, definitive management of the ligamentous knee dislocation ranged from nonoperative approaches to immediate surgical treatment. Notably, an earlier systematic review of literature revealed that immediate surgical management led to increased anterior knee instability, flexion deficits, and joint stiffness compared to delayed reconstruction [22]. Similarly, all three patients in the cases reviewed, who underwent immediate knee ligament reconstruction, required additional procedures for arthrofibrosis, suggesting that delayed reconstruction should be favored.

Therefore, to facilitate effective repair, we recommend following the approach set by the current case and obtaining an MRI during inpatient admission. This allows for assessing the need for prompt surgical intervention determined by involvement of the collateral ligaments, where a compelling argument can be made supporting immediate surgical intervention given the potential inability to achieve a stable concentric reduction with bracing and the high risk potential for vascular compromise. Alternatively, if such urgency is not indicated, a delayed reconstruction strategy is suggested, which has demonstrated superior long-term clinical outcomes and reduces the overall immediate surgical insult on the patient [22].

Only half of the cases reviewed, including the one described here, reported concomitant orthopedic injuries. This is best attributed to the selective force transfer mechanism, isolated to the femur to produce the injury being described. The concomitant injuries posed unique challenges to adequate early rehabilitation in our case. Similarly, the rehabilitation protocol for each case is dependent upon associated injuries but should focus on early mobilization, increasing independence in functional transfers and activities of daily living, and obtaining early range of motion before subsequent orthopedic procedures as seen in this case.

In conclusion, outcomes for the rare injury on an ipsilateral hip and knee dislocation can greatly differ, ranging from complete recovery to limb amputation. This case report underscores the multifaceted approach required for managing such complex orthopedic injuries. Successful management demands a combination of acute stabilization with timely diagnoses, appropriately timed surgical interventions, and focused rehabilitation.

## Disclosures

The authors have received nothing of value in the preparation of this manuscript. There are no conflicts of interest relating to these topics.

## CRediT authorship contribution statement

**Christian G. Falgons:** Writing – review & editing, Writing – original draft, Validation, Resources, Methodology, Investigation, Formal analysis, Data curation. **Stephen J. Warner:** Writing – review & editing, Validation, Supervision, Methodology, Investigation, Conceptualization.

## Declaration of competing interest

The authors declare that they have no known competing financial interests or personal relationships that could have appeared to influence the work reported in this paper.
